# Association of Brain Magnetic Resonance Imaging Signs With Cognitive Outcomes in Persons With Nonimpaired Cognition and Mild Cognitive Impairment

**DOI:** 10.1001/jamanetworkopen.2019.3359

**Published:** 2019-05-10

**Authors:** Aozhou Wu, A. Richey Sharrett, Rebecca F. Gottesman, Melinda C. Power, Thomas H. Mosley, Clifford R. Jack, David S. Knopman, B. Gwen Windham, Alden L. Gross, Josef Coresh

**Affiliations:** 1 Johns Hopkins Bloomberg School of Public Health, Baltimore, Maryland; 2 George Washington University, Washington, DC; 3 University of Mississippi Medical Center, Jackson; 4 Mayo Clinic, Rochester, Minnesota

## Abstract

**Importance:**

Brain atrophy and vascular lesions contribute to dementia and mild cognitive impairment (MCI) in clinical referral populations. Prospective evidence in older general populations is limited.

**Objective:**

To evaluate which magnetic resonance imaging (MRI) signs are independent risk factors for dementia and MCI.

**Design, Setting, and Participants:**

This population-based cohort study included 1553 participants sampled from the Atherosclerosis Risk in Communities Study who had brain MRI scans and were dementia free during visit 5 (June 2011 to September 2013). Participants’ cognitive status was evaluated through visit 6 (June 2016 to December 2017).

**Exposures:**

Brain regional volumes, microhemorrhages, white matter hyperintensity (WMH) volumes, and infarcts measured on 3-T MRI.

**Main Outcomes and Measures:**

Cognitive status (dementia, MCI, or nonimpaired cognition) was determined from in-person evaluations. Dementia among participants who missed visit 6 was identified via dementia surveillance and hospital discharge or death certificate codes. Cox proportional hazards models were used to evaluate the risk of dementia in 3 populations: dementia-free participants (N = 1553), participants with nonimpaired cognition (n = 1014), and participants with MCI (n = 539). Complementary log-log models were used for risk of MCI among participants with nonimpaired cognition who also attended visit 6 (n = 767). Models were adjusted for demographic variables, apolipoprotein E ε4 alleles, vascular risk factors, depressive symptoms, and heart failure.

**Results:**

Overall, 212 incident dementia cases were identified among 1553 participants (mean [SD] age at visit 5, 76 [5.2] years; 946 [60.9%] women; 436 [28.1%] African American) with a median (interquartile range) follow-up period of 4.9 (4.3-5.2) years. Significant risk factors of dementia included lower volumes in the Alzheimer disease (AD) signature region, including hippocampus, entorhinal cortex, and surrounding structures (hazard ratio [HR] per 1-SD decrease, 2.40; 95% CI, 1.89-3.04), lobar microhemorrhages (HR, 1.90; 95% CI, 1.30-2.77), higher volumes of WMH (HR per 1-SD increase, 1.44; 95% CI, 1.23-1.69), and lacunar infarcts (HR, 1.66; 95% CI, 1.20-2.31). The AD signature region volume was also consistently associated with both MCI and progression from MCI to dementia, while subcortical microhemorrhages and infarcts contributed less to the progression from MCI to dementia.

**Conclusions and Relevance:**

In this study, lower AD signature region volumes, brain microhemorrhages, higher WMH volumes, and infarcts were risk factors associated with dementia in older community-based residents. Vascular changes were more important in the development of MCI than in its progression to dementia, while AD-related signs were important in both stages.

## Key Points

**Question:**

To what extent are magnetic resonance imaging–measurable brain lesions associated with dementia and mild cognitive impairment (MCI) among older people?

**Findings:**

In this cohort study of 1553 participants, lower volumes in the Alzheimer disease signature region (hippocampus, entorhinal cortex, and surrounding structures), lobar microhemorrhages, and higher white matter hyperintensity volumes were independent risk factors for dementia and MCI. Findings suggested vascular changes were more important in the development of MCI than in its progression to dementia, whereas Alzheimer disease–related signs were important in both stages.

**Meaning:**

Alzheimer disease–related and vascular-specific magnetic resonance imaging signs together may be associated with the further risk of dementia and MCI in an older population.

## Teaser

This cohort study evaluates which Alzheimer disease and vascular-specific magnetic resonance imaging (MRI) signs may be associated with dementia and mild cognitive impairment in older adults.

## Introduction

Magnetic resonance imaging (MRI) signs of brain atrophy, cerebral microhemorrhages, and cerebral small vessel disease are risk factors of dementia. Alzheimer disease (AD) is marked by atrophy in specific brain regions,^1,2^ namely the AD signature region, which includes the hippocampus, parahippocampal cortex, entorhinal cortex, inferior parietal lobule, precuneus, and cuneus.^3^ However, the associations of specific regional atrophy other than the hippocampus with dementia and MCI incidence are less well known. Prior studies^4,5,6^ of patterns of brain atrophy have observed generally fewer individuals for a shorter time for cognitive outcomes. Most studies of progression from MCI to dementia were restricted to clinical referral populations.^7,8^ The knowledge gap is greatest in populations with nonimpaired cognition.

White matter hyperintensities (WMHs) and lacunar infarcts are signs of small vessel disease. They contribute to vascular dementia^9,10,11,12^ but may also be associated with the pathogenesis of AD.^13^ Microhemorrhages, depending on their locations, play roles in both AD-related and vascular-specific pathology in dementia development.^14^ Lobar microhemorrhages are more associated with cerebral amyloid angiopathy, which is associated with AD pathology. Subcortical microhemorrhages, particularly in deep gray and white matter, are more likely to be associated with hypertensive arteriolar disease and, therefore, considered vascular signs.^15^ Alzheimer disease and vascular signs may each have an independent role in dementia progression, but research on their joint effects in general populations is limited.

Using MRI measures and longitudinal determinations of cognitive status (nonimpaired, MCI, or dementia) of participants in the Atherosclerosis Risk in Communities (ARIC) Study, we evaluated the associations of lower volumes as an index of atrophy in the AD signature region, microhemorrhages by location (lobar vs subcortical),^16^ higher volumes of WMH, and infarcts with risk of incident dementia in the dementia-free cohort, risk of dementia and MCI in the cohort with nonimpaired cognition, and risk of progression from MCI to dementia. We studied the prognostic value of AD and vascular signs both separately and jointly.

## Methods

### Study Population

The ARIC study is an ongoing prospective cohort with 15 792 participants (aged 45-64 years) enrolled from December 1986 to January 1990 from 4 US communities. Neurocognitive study (NCS) examinations were conducted at visit 5 (June 2011 to September 2013), the index visit (baseline) for this analysis, and at visit 6 (June 2016 to December 2017). Among the 6538 participants who attended visit 5 (aged 66-90 years), a selected sample of 1978 participants, enriched for cognitive impairment, completed a brain MRI scan, as documented previously.^12,17^ We excluded participants with prevalent dementia at the index visit, nonwhite and non–African American participants, and those missing MRI signs of interest, cognitive measurements, or covariates, leaving 1553 participants for this study (eFigure 1 in the Supplement). In the analysis for MCI, we excluded participants who had MCI at visit 5 (n = 539) or did not attend visit 6 (n = 247), because MCI could be diagnosed only at an examination, leaving 767 participants. All participants gave informed written consent, and the study was approved by the institutional review board at each study site. This study followed Strengthening the Reporting of Observational Studies in Epidemiology (STROBE) reporting guideline.

### Cognitive Outcomes

Cognitive status (nonimpaired, MCI, or dementia) of all participants who attended visit 5 was classified using a standardized algorithm based on cognitive assessment and verified by expert committee review (R.F.G., T.H.M., D.S.K., and B.G.W), using information from in-person cognitive battery scores, the clinical dementia rating scale, and functional questionnaires completed by participants and/or informants, as previously reported.^17^ Participants who attended visit 6 were similarly classified, based on the same criteria. For participants who missed visit 6, incident dementia (but not MCI) was identified from dementia surveillance using annual follow-up telephone interviews and hospital discharge and death certificate codes (eMethods and eTable 1 in the Supplement). Incident dementia was identified through December 31, 2017. When dementia was diagnosed, its onset was the earliest date indicating dementia, determined at the visit 6 examination, by dementia surveillance, or by hospital discharge or death certificate codes.

### Brain Imaging

Structural brain images were obtained using 3-T MRI scanners (Siemens Verio [Maryland study center], Siemens Skyra [North Carolina study center], Siemens Trio [Minnesota study center], and Siemens Skyra [Mississippi study center]). Details were described previously.^16^ Brain volumes were estimated based on T1-weighted scans. Volumes of regions of interest were estimated using the FreeSurfer system (Laboratory for Computational Neuroimaging). Specifically, we evaluated the volume of total brain cortical region, hippocampus, and other AD signature regions, including parahippocampal, entorhinal, inferior parietal lobule, precuneus, and cuneus.^15^ White matter hyperintensity volume was measured using a semiautomated segmentation algorithm^18^ on T2-weighted fluid attenuation inversion recovery images. Brain microhemorrhages and infarcts were identified by a trained imaging technician and confirmed by a radiologist (C.R.J.). Depending on the location, microhemorrhages were further classified as lobar (at lobar or cortical gray) or subcortical (subcortical or periventricular)^16^ microhemorrhages on T2-weighted images; infarcts were further classified as lacunar or cortical infarcts on T2-weighted fluid attenuation inversion recovery images. Details are described in the eMethods in the Supplement.

### Covariates

We included potential confounders^19^ in the model, including age, sex, race, education level (ie, <high school, high school or equivalent, ≥college), apolipoprotein E (*APOE*) ε4 allele genotype (0 or ≥1 alleles), self-reported cumulative smoking history (pack-years), body mass index (calculated as weight in kilograms divided by height in meters squared), total cholesterol level (milligrams per deciliter), hypertensive status (yes or no, defined as use of hypertension medication, systolic blood pressure >140 mm Hg, or diastolic blood pressure >90 mm Hg), diabetes (yes or no, defined as self-reported diabetes diagnosis by physicians, use of diabetes medication, or having hemoglobin A_1c_ level of ≥6.5% [to convert to proportion of total hemoglobin, multiply by 0.01]), history of heart failure,^20^ and depressive symptoms (defined using the 11-item Center for Epidemiologic Studies Depression Scale, with a cutoff score of ≥9).^21^ All covariates reflect participant status at the index visit. We included prevalent stroke as a covariate in a sensitivity analysis. Total intracranial volume was included in models for volume-based exposures.

### Statistical Analysis

The baseline characteristics of participants were compared by cognitive status (nonimpaired vs MCI) at the index visit and analyzed using the χ^2^ test or 2-sample *t* test, as appropriate. We studied 4 primary cognitive outcomes: (1) incident dementia among participants who were dementia free at the index visit, (2) incident dementia among those who had no cognitive impairment, (3) incident MCI among participants who had no cognitive impairment, and (4) incident dementia among those who had MCI at the index visit.

Volume measurements, including volumes of the AD signature region and WMH, were standardized by population means and standard deviations and modeled as continuous variables. White matter hyperintensity volume was log transformed before the standardization to account for skewness. When studying the relative association of the number of AD-related signs vs number of vascular-related signs (Figure and eFigure 2 in the Supplement), dichotomized volume measurements were used, defining a low volume in the hippocampus and nonhippocampal AD signature regions as a value lower than the lowest quartile and a high WMH volume as a value higher than the median. Brain infarcts and microhemorrhages were modeled as binary exposures, ie, any vs none.

**Figure.**
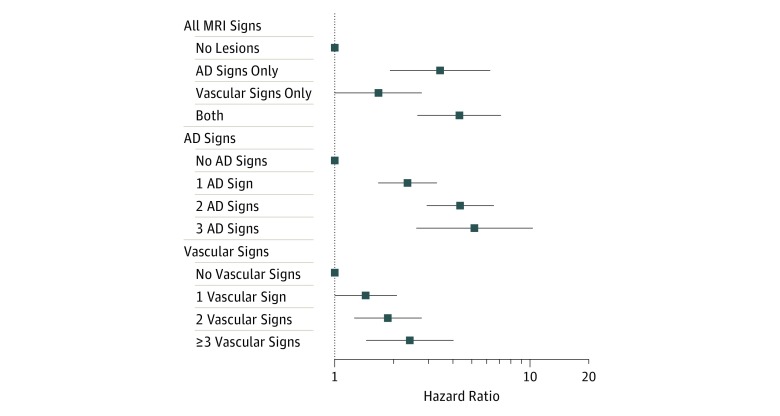
Dementia Risk by Number of Different Types of Magnetic Resonance Imaging (MRI) Signs Hazard ratios and 95% CIs of incident dementia in the dementia-free population were plotted. Model adjusted for age, sex, race, education, apolipoprotein E ε4 allele, smoking, body mass index, hypertension, diabetes, cholesterol, and heart failure. The MRI signs of interest were 3 Alzheimer disease (AD)–related signs: (1) low hippocampus volume, (2) low nonhippocampal AD signature region volume (1 and 2 defined as a value lower than the lowest quartile), and (3) having lobar microhemorrhages; and 4 vascular signs: (1) high white matter hyperintensity volume (defined as a value higher than the median), (2) having subcortical microhemorrhages, (3) having cortical infarcts, and (4) having lacunar infarcts. The number of different MRI signs was modeled as a categorical variable. The categories of no lesions, no AD signs, and no vascular signs were considered reference categories.

Primary analyses on incident dementia used Cox proportional hazard models with Efron method to handle ties. We checked the proportional hazards assumption and collinearity. The analysis for incident MCI used complementary log-log models, which treated the data as interval censored. The primary model included all the aforementioned covariates. Magnetic resonance imaging signs were modeled separately, meaning 1 type of lesion sign per model, and jointly, meaning including several types of lesion in a single model. Adjusted hazard ratios (HRs) and 95% CIs were calculated. In sensitivity analyses, we confirmed all results using logistic regression. We also further adjusted for prevalent stroke. Results from sensitivity analyses were not shown. The prognostic value of different MRI signs was evaluated for 6-year dementia risk prediction using Somers *D* statistics,^22^ which reflects the prediction model discriminative accuracy and is equivalent to C statistics in the survival setting. Models with MRI signs were compared with the base model using the *t* test for prediction improvement in the entire analytical population.^23^ Prediction performance was further validated using 5-fold cross-validation (eMethods in the Supplement). *P* < .05 was considered statistically significant for all analyses, and tests were 2-tailed. All statistical analyses were conducted using Stata version 14.2 (StataCorp).

## Results

### Participant Characteristics

We included 1553 ARIC participants who had brain MRI measurements and were free of dementia at the index visit. The mean (SD) age was 76 (5.2) years. There were 946 (60.9%) women, 436 (28.1%) African American individuals, and 539 participants (34.7%) with MCI at the index visit. We identified 212 incident dementia cases through a median (interquartile range) follow-up of 4.9 (4.3-5.2) years, and 114 newly developed MCI cases among 767 participants with nonimpaired cognition at visit 5 who attended visit 6. Compared with those who had no cognitive impairment at baseline, participants with MCI were older, more likely to be men, less educated, and had *APOE* ε4 alleles and prevalent heart failure (Table 1). Compared with the entire ARIC population at visit 5, we did not find meaningful differences in the subpopulation with MRI measurements.

**Table 1.** Characteristics and Dementia Incidence by Cognitive Status at the Index Visit in 2011-2013

Characteristic	No. (%)			*P* Value^a^
	Total (N = 1553)	Participants With Normal Cognition (n = 1014)	Participants With MCI (n = 539)	
Age, mean (SD), y	76.0 (5.2)	75.8 (5.3)	76.5 (5.2)	.02
Women	946 (60.9)	642 (63.3)	304 (56.4)	.008
African American	436 (28.1)	320 (31.6)	116 (21.5)	<.001
Education				
<High school	199 (12.8)	131 (12.9)	68 (12.6)	.003
High school	644 (41.5)	390 (38.5)	254 (47.1)	
≥College	710 (45.7)	493 (48.6)	217 (40.3)	
*APOE* ε4 status	437 (28.1)	263 (25.9)	174 (32.3)	.008
Smoking history, pack-years				
Never	671 (43.2)	445 (43.9)	226 (41.9)	.34
<25	593 (38.2)	391 (38.6)	202 (37.5)	
≥25	289 (18.6)	178 (17.6)	111 (20.6)	
Body mass index, mean (SD)^b^	28.4 (5.6)	28.4 (5.6)	28.5 (5.7)	.63
Total cholesterol level, mean (SD), mg/dL	185.6 (42.5)	185.6 (42.5)	181.8 (42.5)	.11
Hypertension	1163 (74.9)	750 (74.0)	413 (76.6)	.25
Diabetes	500 (32.2)	317 (31.3)	183 (34.0)	.28
Heart failure	156 (10.0)	89 (8.8)	67 (12.4)	.02
Stroke	52 (3.3)	29 (2.9)	23 (4.3)	.14
Depressive symptoms	104 (6.7)	60 (5.9)	44 (8.2)	.09
Incident dementia	212 (13.7)	70 (6.9)	142 (26.3)	<.001

Abbreviation: *APOE*, apolipoprotein E ; MCI, mild cognitive impairment.

SI conversion factor: To convert total cholesterol to millimoles per liter, multiply by 0.0259.

^a^ Differences between participants with no cognitive impairment and MCI were compared using the χ^2^ test for binary and categorical variables and the 2-sample *t* test for continuous variables.

^b^ Calculated as weight in kilograms divided by height in meters squared.

### Association of AD Signature Region Volume With Dementia and MCI

Participants with smaller AD signature region volumes had a higher risk of incident dementia with an HR per 1-SD decrease of 2.40 (95% CI, 1.89-3.04). Similar associations were seen for the hippocampus (HR per 1-SD decrease: 1.67; 95% CI, 1.50-1.86) and nonhippocampal AD signature regions (HR per 1-SD decrease: 2.07; 95% CI, 1.63-2.62), independent of common dementia risk factors (Table 2). These associations of volume with dementia were approximately linear (eFigure 3A and B in the Supplement).

**Table 2.** Hazard Ratios of Incident Dementia for Different MRI Signs

Brain MRI Sign	MRI Sign Summary Statistic	Incident Dementia^a^	
		HR (95% CI)^b^	*P* Value
**AD-Related Atrophy Signs**			
Lower brain volume, mean (SD), cm^3^^c^			
AD signature region volume	59.35 (6.84)	2.40 (1.89-3.04)	<.001
Hippocampus volume	6.92 (1.02)	1.67 (1.50-1.86)	<.001
Nonhippocampal AD signature region volume	52.43 (6.29)	2.07 (1.63-2.62)	<.001
Brain microhemorrhages, No. (%)			
Microhemorrhages (any)	356 (22.9)	1.27 (0.94-1.70)	.11
Lobar microhemorrhages	131 (8.4)	1.90 (1.30-2.77)	.001
Subcortical microhemorrhages	295 (19.0)	1.32 (0.97-1.79)	.08
**Vascular Signs**			
Log WMH volume, mean (SD), cm^3c^	16.67 (16.40)	1.44 (1.23-1.69)	<.001
Brain infarcts, No. (%)			
Any infarcts	375 (24.1)	1.60 (1.19-2.16)	.002
Cortical infarcts	151 (9.7)	1.18 (0.78-1.80)	.43
Lacunar infarcts	259 (16.7)	1.66 (1.20-2.31)	.002

Abbreviations: AD, Alzheimer disease; HR, hazard ratio; MRI, magnetic resonance imaging; WMH, white matter hyperintensity.

^a^ Of 1553 participants, 212 had incident dementia after visit 5.

^b^ Hazard ratio of incident dementia in the dementia-free population at the index visit (June 2011 to September 2013) were estimated using separate models (1 MRI sign per model), adjusted for age, sex, race, education, apolipoprotein E ε*4 *allele, smoking, body mass index, hypertension, diabetes, total cholesterol level, depressive symptoms, and heart failure. In models assessing volume-based measurements, we further adjusted for intracranial volume.

^c^ Hazard ratios estimated using standardized volume measurements with results presented per 1-SD decrease for AD-related atrophy signs and per 1-SD increase for WMH volume.

Examining subpopulations by cognitive status, lower AD signature region volumes were associated with incident dementia (HR per 1-SD decrease, 2.59; 95% CI, 1.69-3.96) and MCI (HR per 1-SD decrease, 1.71; 95% CI, 1.24-2.34) among the subpopulation with nonimpaired cognition as well as progression from MCI to dementia (HR per 1-SD decrease, 1.92; 95% CI, 1.42-2.60). Similar effects were seen for hippocampal and nonhippocampal volumes (Table 3). Of note, total brain volume was also associated with dementia risk. However, that association was no longer significant when adjusting for the AD signature region volume and other signs (WMH, infarcts, and microhemorrhages) (eTable 2 in the Supplement).

**Table 3.** Hazard Ratios of Cognitive Outcomes for Different MRI Signs by Cognitive Status at Index Visit in 2011-2013

Brain MRI Sign	Participants With Nonimpaired Cognition				Participants With MCI	
	Incident Dementia^a^		Incident MCI^a^		Incident Dementia^a^	
	HR (95% CI)^b^	*P* Value	HR (95% CI)^c^	*P* Value	HR (95% CI)^b^	*P* Value
**AD-Related Atrophy Signs**						
Lower brain volume^d^						
AD signature region volume	2.59 (1.69-3.96)	<.001	1.71 (1.24-2.34)	.001	1.92 (1.42-2.60)	<.001
Hippocampus volume	1.72 (1.42-2.09)	<.001	1.27 (1.09-1.48)	.002	1.54 (1.32-1.80)	<.001
Nonhippocampal AD signature region volume	2.22 (1.46-3.38)	<.001	1.56 (1.14-2.14)	.005	1.73 (1.27-2.35)	.001
Brain microhemorrhages						
Any microhemorrhages	1.57 (0.95-2.60)	.08	1.79 (1.19-2.68)	.005	0.99 (0.68-1.44)	.95
Lobar microhemorrhages	1.67 (0.81-3.45)	.17	1.83 (1.02-3.28)	.04	1.69 (1.08-2.67)	.02
Subcortical microhemorrhages	1.56 (0.92-2.64)	.10	1.62 (1.04-2.51)	.03	1.09 (0.74-1.60)	.66
**Vascular Signs**						
Log WMH volume^d^	1.44 (1.08-1.93)	.01	1.11 (0.90-1.38)	.33	1.30 (1.07-1.58)	.008
Brain infarcts						
Any infarcts	2.86 (1.73-4.70)	<.001	1.35 (0.87-2.08)	.18	1.07 (0.74-1.56)	.72
Cortical infarcts	1.89 (0.97-3.67)	.06	1.32 (0.72-2.45)	.37	0.78 (0.45-1.36)	.38
Lacunar infarcts	2.79 (1.63-4.78)	<.001	1.58 (0.97-2.56)	.06	1.14 (0.75-1.72)	.54

Abbreviations: AD, Alzheimer disease; HR, hazard ratio; MCI, mild cognitive impairment; MRI, magnetic resonance imaging; WMH, white matter hyperintensity.

^a^ Of 1014 participants with nonimpaired cognition, 70 had incident dementia after visit 5. In the analysis of incident MCI, 247 participants were excluded because they did not have a cognitive assessment at visit 6 (June 2016 to December 2017). Of the remaining 767 participants, 114 had incident MCI after visit 5. Of 539 participants with MCI, 142 had incident dementia after visit 5.

^b^ Hazard ratio of incident dementia in the dementia-free population at the index visit (2011-2013) were estimated using separate models (1 MRI sign per model), adjusted for age, sex, race, education, apolipoprotein E ε4 allele, smoking, body mass index, hypertension, diabetes, total cholesterol level, depressive symptoms, and heart failure. In models assessing volume-based measurements, we further adjusted for intracranial volume.

^c^ Complementary log-log models were used to model the HR.

^d^ Hazard ratios estimated using standardized volume measurements with results presented per 1-SD decrease for AD-related atrophy signs and per 1-SD increase for WMH volume.

### Associations of Microhemorrhages With Dementia

Microhemorrhages were not associated with dementia risk in the dementia-free population (HR, 1.27; 95% CI, 0.94-1.70). They were significantly associated with incident MCI (HR, 1.79; 95% CI, 1.19-2.68). Of note, a significant association was seen for lobar microhemorrhages with incident dementia (HR, 1.90; 95% CI, 1.30-2.77), stronger than for subcortical microhemorrhages (HR, 1.32; 95% CI, 0.97-1.79) (Table 2). Lobar microhemorrhages were also associated with a 1.8-fold risk of incident MCI and 1.7-fold risk of progression from MCI to dementia. In contrast, subcortical microhemorrhages were associated with MCI in participants with nonimpaired cognition but not with incident dementia or the progression from MCI to dementia (Table 3).

### Association of WMHs and Infarcts With Dementia Risk

Higher WMH volumes were associated with an elevated risk of dementia in the dementia-free population (HR per 1-SD increase, 1.44; 95% CI, 1.23-1.69), in what might be a threshold manner (Table 2 and eFigure 3C in the Supplement). The association of WMH volume with dementia was largely consistent in participants with nonimpaired cognition and participants with MCI, while its association with incident MCI was not significant (Table 3).

Infarcts demonstrated an independent association with incident dementia (HR, 1.60; 95% CI, 1.19-2.16), particularly lacunar infarcts (HR, 1.66; 95% CI, 1.20-2.31). Cortical infarcts were not associated with dementia (HR, 1.18; 95% CI, 0.78-1.80) (Table 2). Infarcts, particularly lacunar infarcts, were also associated with incident dementia among participants with nonimpaired cognition but were not associated with incident MCI or the progression from MCI to dementia (Table 3).

### Combined Associations of AD-Related and Vascular MRI Signs

Magnetic resonance imaging signs were grouped as AD related (atrophy and lobar microhemorrhages) and vascular related (WMH, infarcts, and subcortical microhemorrhages) to study their separate and joint associations. The hippocampus volume, nonhippocampal AD signature region volume, and lobar microhemorrhages were independently associated with dementia when modeled together (Table 4). When vascular signs were modeled together, we observed an independent association of WMH volume with incident dementia, but there was no association of other vascular signs with incident dementia. Taking all signs together in the same model, the 3 AD-related signs, ie, hippocampus volume, nonhippocampal region volume, and lobar microhemorrhages, and 1 vascular sign, ie, WMH volume, remained significant with little change in their HRs (Table 4). Overall, having either AD-related or vascular-related signs was associated with incident dementia, but the largest HR was seen in those with both types of lesions together (HR, 4.34; 95% CI, 2.65-7.10) (Figure). A roughly linear association was seen between the number of AD-related signs and dementia risk (1 AD sign: HR, 2.36; 95% CI, 1.67-3.34; 2 AD signs: HR, 4.39; 95% CI, 2.96-6.53; 3 AD signs: HR, 5.21; 95% CI, 2.62-10.34). A similar but weaker dose-response association was seen with risk of dementia and a number of different vascular signs (1 vascular sign: HR, 1.44; 95% CI, 1.00-2.08; 2 vascular signs: HR, 1.87; 95% CI, 1.26-2.79; ≥3 vascular signs: HR, 2.43; 95% CI, 1.45-4.06) (Figure). Similar but somewhat weaker associations were seen for the group with MCI at baseline. The association of vascular signs and risk of dementia among participants with MCI was not significant (eFigure 2 in the Supplement).

**Table 4.** Association of AD-Related and Vascular Signs With Incident Dementia When Modeled Simultaneously

Brain MRI Sign	AD-Related		Vascular		AD-Related and Vascular	
	HR (95% CI)^a^	*P* Value	HR (95% CI)^a^	*P* Value	HR (95% CI)^a^	*P* Value
**AD-Related Signs**						
Hippocampus volume^b^	1.61 (1.43-1.82)	<.001	NA	NA	1.63 (1.44-1.85)	<.001
Nonhippocampal AD signature region volume^b^	1.62 (1.28-2.06)	<.001	NA	NA	1.57 (1.24-2.00)	<.001
Lobar microhemorrhages	1.92 (1.31-2.80)	.001	NA	NA	1.58 (1.03-2.41)	.03
**Vascular Signs**						
Log WMH volume^b^	NA	NA	1.38 (1.17-1.63)	<.001	1.29 (1.09-1.53)	.003
Cortical infarcts	NA	NA	1.05 (0.68-1.60)	.84	0.97 (0.63-1.48)	.89
Lacunar infarcts	NA	NA	1.37 (0.98-1.93)	.06	1.36 (0.96-1.92)	.09
Subcortical microhemorrhages	NA	NA	1.16 (0.84-1.58)	.37	1.02 (0.73-1.44)	.90

Abbreviations: AD, Alzheimer disease; HR, hazard ratio; MRI, magnetic resonance imaging; NA, not applicable; WMH, white matter hyperintensity.

^a^ Hazard ratio of incident dementia in the dementia-free population (N = 1553) at the index visit (2011-2013), adjusted for age, sex, race, education, apolipoprotein E ε4 allele, smoking, body mass index, hypertension, diabetes, total cholesterol level, depressive symptoms, and heart failure. Intracranial volume for volume measurements.

^b^ Standardized volume measurements with results presented per 1-SD decrease for AD-related atrophy signs and per 1-SD increase for WMH volume.

### Sensitivity Analysis and Prediction Performance

The sensitivity analyses using logistic regression provided similar results for the aforementioned associations. The further adjustment for stroke as another sensitivity analysis did not change the results.

Magnetic resonance imaging signs have additional prognostic value compared with traditional dementia risk factors, including age, sex, race, education, *APOE* ε4 allele, smoking, body mass index, hypertension, diabetes, total cholesterol level, and heart failure, which composed our base prediction model. Adding both AD and vascular signs to the base model significantly improved prediction accuracy for 6-year dementia risk measured by Somers *D* statistics from 0.768 (95% CI, 0.739-0.799) to 0.811 (95% CI, 0.781-0.840; *P* < .001) (eTable 3 in the Supplement). The 5-fold cross-validation showed an improvement in Somers *D* from 0.748 to 0.792.

## Discussion

In this community-living sample, we found lower volumes of AD signature regions were associated with incident dementia and MCI as well as the progression from MCI to dementia. Having higher WMH volumes, lobar microhemorrhages, and lacunar infarcts were also risk factors. Their associations were largely independent of each other.

Volumes of both the hippocampus and the nonhippocampal AD signature regions were risk factors of dementia, independent of each other, in both the population with nonimpaired cognition and those with MCI. This is consistent with published evidence from clinical referral populations^7,24,25,26,27,28^ and a population-based study with only a few events.^29^ Similar associations have also been reported in a population with MCI.^30^ Together these results suggest that the prognostic value of AD signature region volume is important in early and late stages of dementia progression. Of note, total brain volume was a substantial risk factor of dementia, as has been previously reported.^5,6^ However, its association can be largely explained by the volume of AD signature regions.

Our community-based study largely agrees with previous evidence of the associations of WMH,^12,31,32^ infarcts,^12,32,33^ and microhemorrhages^34,35,36,37^ with dementia. We examined subtypes of the signs. For example, lacunar infarcts, which are located in the subcortical regions, were more strongly associated with dementia than the much larger cortical infarcts. Lobar microhemorrhages, a sign of cerebral amyloid angiopathy, which in turn has been found to be associated with parenchymal AD pathology, showed a stronger association with dementia than subcortical microhemorrhages, which are more associated with hypertensive arteriolar lesions.^15^ This implies a greater role of AD-related pathology than vascular-specific pathology in dementia development in this community-based older population.

When looking at subpopulations classified by baseline cognitive status, we found that infarcts were associated with dementia and MCI incidence in the population with nonimpaired cognition but not with progression from MCI to dementia. However, lobar microhemorrhages, which are AD related, and AD signature region atrophy retained an association with the progression from MCI to dementia. These findings suggest that infarcts might play an earlier role in dementia development, while AD-related lesions may dominate the progress at a later stage. This inference is supported by a report from the Honolulu Asia Aging Study^38^ and implies, as the authors stated, that vascular changes occur relatively early in the trajectory to dementia, with AD tangles at a later stage.

The pathology of AD and of cerebrovascular diseases often overlap.^39^ Of note, our results suggest that the associations of AD-related signs and vascular signs with dementia are independent of each other. In our study, including AD signature region volumes did not substantially attenuate the associations of the vascular lesions with dementia. A clear cumulative association was seen for a number of different types of MRI lesions when considering AD-related signs and vascular signs together. Potentially, atrophy in AD signature regions and lobar microhemorrhages captured the pathologies more related to AD development,^3,15^ while the other lesions may have comprised the cerebrovascular contribution to cognitive impairment.^40^ Previous studies^41,42^ used demographic information, *APOE* allele type, and major vascular comorbidities to predict dementia, with similar performance as our base prediction model using similar information. Our results show that adding MRI lesion signs improved prediction accuracy. Associations are also seen for MCI incidence and progression from MCI to dementia. This implies that, compared with general risk factors of dementia, brain imaging signs may better assist a clinician in estimating an elderly patient’s prognosis for remaining cognitively healthy.

### Strengths and Limitations

The current study has several strengths. It uses including brain imaging data with longitudinal cognitive assessment in a large community-based older population, including individuals with MCI. Comprehensive cognitive assessments and consistent definitions were used to define dementia and MCI at 2 study visits 5 years apart. The dementia surveillance system captured dementia cases occurring among participants who did not come to visit 6, therefore minimizing the potential for bias from loss to follow-up. We evaluated AD-related signs and vascular signs separately and jointly to study their independent associations. We also examined these associations in different stages of dementia development.

However, limitations exist. Although 1-time measurement of brain volume relative to intracranial volume has been previously used to estimate the degree of brain atrophy,^32^ we cannot estimate the true scale or rate of atrophy. We only studied dementia onset without exploring cognitive decline as a continuous measurement. We also did not further differentiate the cases of AD and vascular dementia. Our dementia surveillance captured dementia but not MCI incidence among individuals who missed examination at visit 6. In the analysis of MCI risk, we excluded those who missed visit 6. By doing so, our results might be biased but are likely attenuated toward the null owing to differential cohort attrition, because participants with poorer cognitive function and brain lesion signs are more likely to be lost to follow-up. For the MCI study, we did not have a good estimate of the time of MCI onset. Logistic regression, which does not require time of onset, was used in a sensitivity analysis and replicated the results.

## Conclusions

In this study, MRI signs, including AD-related signs and signs of cerebral small vessel disease, were independent risk factors for dementia in older age. Smaller volumes in AD signature regions, having lobar microhemorrhages, and higher WMH volumes were consistently associated with dementia. Our findings suggest the potential importance of both AD-related and vascular-specific signs in the development of dementia and MCI in older populations, even among those who have nonimpaired cognition, as well as in progression from MCI to dementia.

## Notes

Supplement. **eMethods**. Supplemental Methods **eTable 1.** *ICD*-*9* and* ICD-10* Codes for Dementia Diagnosis **eTable 2.** The Attenuation in the Association of Total Brain Volume With Dementia Risk After Adjusting for Other MRI Signs **eTable 3.** Prediction Accuracy of 6-Year Dementia Risk Prediction Using MRI Signs **eFigure 1.** Study Population Flow **eFigure 2.** Dementia Risk by Number of Different Types of MRI Signs Among Participants With MCI **eFigure 3.** Adjusted Hazard Ratio of Incident Dementia Across Volumes of AD Signature Regions and WMH
