# Supplementary material for: Association of Brain Magnetic Resonance Imaging Signs With Cognitive Outcomes in Persons With Nonimpaired Cognition and Mild Cognitive Impairment
Source: JAMA Netw Open. 2019 May 10;2(5):e193359. doi: 10.1001/jamanetworkopen.2019.3359 (PMC6512274; doi:10.1001/jamanetworkopen.2019.3359)
Supplement: Supplement. — eMethods. Supplemental Methods eTable 1. ICD-9 and ICD-10 Codes for Dementia Diagnosis eTable 2. The Attenuation in the Association of Total Brain Volume With Dementia Risk After Adjusting for Other MRI Signs eTable 3. Prediction Accuracy of 6-Year Dementia Risk Prediction Using MRI Signs eFigure 1. Study Population Flow eFigure 2. Dementia Risk by Number of Different Types of MRI Signs Among Participants With MCI eFigure 3. Adjusted Hazard Ratio of Incident Dementia Across Volumes of AD Signature Regions and WMH [file jamanetwopen-2-e193359-s001.pdf]

# Supplementary Online Content

Wu A, Sharrett AR, Gottesman RF, et al. Association of brain magnetic resonance imaging signs with cognitive outcomes in persons with nonimpaired cognition and mild cognitive impairment. *JAMA Netw Open*. 2019;2(5):e193359. doi:10.1001/jamanetworkopen.2019.3359

**eMethods.** Supplemental Methods

**eTable 1.** *ICD-9* and *ICD-10* Codes for Dementia Diagnosis

**eTable 2.** The Attenuation in the Association of Total Brain Volume With Dementia Risk After Adjusting for Other MRI Signs

**eTable 3.** Prediction Accuracy of 6-Year Dementia Risk Prediction Using MRI Signs

**eFigure 1.** Study Population Flow

**eFigure 2.** Dementia Risk by Number of Different Types of MRI Signs Among Participants With MCI

**eFigure 3.** Adjusted Hazard Ratio of Incident Dementia Across Volumes of AD Signature Regions and WMH

This supplementary material has been provided by the authors to give readers additional information about their work.

© 2019 Wu A et al. *JAMA Network Open.*

## eMethods. Supplemental Methods

### Dementia Identification Among Participants Who Missed Visit 6

Dementia status (dementia vs. non-dementia) for participants who missed visit 6 in-person examination were determined using two data sources: 1) dementia surveillance using annual follow-up telephone interviews and 2) dementia hospital discharge codes and death certificate codes.

Cognition information was collected from participants and informers through dementia surveillance system and used to determine the dementia status of participants who missed visit 6 in-person examination and the earliest dementia onset date for those identified as incident dementia case. Dementia surveillance based on annual follow-up telephone interviews was initiated after visit 5. This consisted of two screening tools: the six-item screener (SIS)[1] which is a brief instrument derived from Mini-Mental State Examination, and the AD8[2] which is an informant-based tool derived from the Clinical Dementia Rating. The SIS was administrated to all participants who were able to answer questions by phone and has a sensitivity of 89% and specificity of 88% for diagnosing dementia using a score cut-off of less than or equal to three[1]. The AD8 was administered to the proxies of participants when 1) there was an impaired SIS (a total score <= 2 or failure on all 3 orientation items) on a previous annual interview; 2) the SIS could not be administered, e.g., due to hearing loss; 3) a proxy was needed for the annual phone interview; or 4) the participant was deceased and no prior AD8 had been obtained. The specific criteria for dementia included a failed AD8, defined as a score $\geq 2$, or two failed SIS with SIS scores $\leq 3$.

For participants who had neither an in-person evaluation at a study visit nor available dementia surveillance data, dementia status was determined based on ICD-9 and ICD-10 dementia diagnosis codes (**eTable 1**) obtained from hospital records and death certificates from cohort surveillance inception.

### Definition of Micro-hemorrhages and Infarcts

Brain images were reviewed by a trained imaging technician and confirmed by a radiologist (Dr. Kejal Kantarci or Dr. Clifford R. Jack Jr) to identify brain micro-hemorrhages and infarcts. Micro-hemorrhages were defined as homogeneous black lesions with round or ovoid shapes on T2*-weighted MRI and blooming effect on GRE sequences. Depending on the location, they were further classified as lobar (at lobar or cortical gray) or subcortical micro-hemorrhages (subcortical or periventricular)[3]. Infarcts were hyperintense lesions on T2 FLAIR images. Particularly, lacunar infarcts, a particular type of subcortical infarcts were shown as spots with central hypointensity > 3 mm and hyperintensity < 15 mm in diameter in the white matter, infratentorial, and central gray/capsular regions, and distinguishable from perivascular spaces. Cortical infarctions were T2 FLAIR lesions located in cortical surfaces.

### Prediction Model Details

We first evaluated the prediction performance of the MRI signs using the entire analytical sample. The base prediction model included predictors: age, gender, race, education, APOE ε4 alleles, smoking, body mass index, hypertension, diabetes, total cholesterol level, and heart failure. Marginal improvement, calculated as the changes in Somers' D, after including one or multiple MRI signs as compared to the base model, was tested for significance using the t-test. In addition, we validated the prediction model performance using a 5-fold cross-validation. An averaged Somers' D statistics across the 5 validation subsets was reported for each model without a further statistical test.

### Reference


1. Callahan CM, Unverzagt FW, Hui SL, Perkins AJ and Hendrie HC. Six-item screener to identify cognitive impairment among potential subjects for clinical research. *Medical care*. 2002;40:771-81.
2. Galvin JE, Roe CM, Powlishta KK, Coats MA, Muich SJ, Grant E, Miller JP, Storandt M and Morris JC. The AD8: a brief informant interview to detect dementia. *Neurology*. 2005;65:559-64.
3. Schneider ALC, Selvin E, Sharrett AR, Griswold M, Coresh J, Jack CR, Jr., Knopman D, Mosley T and Gottesman RF. Diabetes, Prediabetes, and Brain Volumes and Subclinical Cerebrovascular Disease on MRI: The Atherosclerosis Risk in Communities Neurocognitive Study (ARIC-NCS). *Diabetes care*. 2017;40:1514-1521.


© 2019 Wu A et al. *JAMA Network Open.*

## eTable 1. *ICD-9* and *ICD-10* Codes for Dementia Diagnosis

| Code Sources | Code [a] |
|---|---|
| ICD-9-CM | Starting with or equal to<br>290 (including: 290.0, 290.1x, 290.2x, 290.3, 290.4x, 290.8, 290.9);<br>294 (including: 294.0, 294.1x, 294.2x, 294.9);<br>331 (including: 331.0, 331.1x, 331.2, 331.7, 331.8x, 331.9; but excluding 331.83 – mild cognitive impairment) |
| ICD-10-CM | Starting with or equal to<br>F01 (including: F01.5x);<br>F02 (including: F02.8x);<br>F03 (including: F03.9x);<br>F04;<br>F06.8;<br>G30 (including: G30.1, G30.8, G30.9);<br>G31 (including: G31.0x, G31.1, G31.8x, G31.9; but excluding G31.84 – mild cognitive impairment);<br>G94<br>R41 (including: R41.8x, R41.9) |

a. Code with a suffix “x” can have one subsequent digit from 0 to 9 in the place of “x” when applicable.

© 2019 Wu A et al. *JAMA Network Open.*

**eTable 2. The Attenuation in the Association of Total Brain Volumes With Dementia Risk After Adjusting for Other MRI Signs [a]**

| Brain MRI Signs | Total brain volume only | | With other atrophy signs | | With all other signs | |
|---|---|---|---|---|---|---|
| | HR (95%CI) | P-value | HR (95%CI) | P-value | HR (95%CI) | P-value |
| *Total brain region volume* [b] | 2.38 (1.77 to 3.22) | <0.001 | 1.21 (0.83 to 1.77) | 0.32 | 1.13 (0.77 to 1.65) | 0.54 |
| *Hippocampus volume* [b] | – | | 1.56 (1.38 to 1.78) | <0.001 | 1.62 (1.42 to 1.84) | <0.001 |
| *Non-hippocampal AD signature region volume* [b] | – | | 1.53 (1.15 to 2.04) | 0.003 | 1.51 (1.14 to 2.02) | 0.004 |
| *Log WMH volume* [b] | – | | – | | 1.32 (1.12 to 1.56) | 0.001 |
| *Infarcts (any)* | – | | – | | 1.37 (1.01 to 1.86) | 0.04 |
| *Micro-hemorrhages (any)* | – | | – | | 1.18 (0.87 to 1.59) | 0.29 |

AD = Alzheimer disease, WMH = white matter hyperintensities
a. Adjusted for: age, gender, race, education, APOE ε4, smoking, body mass index, hypertension, diabetes, total cholesterol level, depressive symptoms, heart failure, and intracranial volume
b. Standardized volume measurements with results presented per 1 SD change.

© 2019 Wu A et al. *JAMA Network Open.*

**eTable 3. Prediction Accuracy of 6-Year Dementia Risk Using MRI Signs**

<table>
<tr><th rowspan="2">Brain MRI Signs</th><th colspan="2">Each Individual Signs</th><th colspan="2">AD-related Signs Only</th><th colspan="2">Vascular Signs Only</th><th colspan="2">AD and Vascular Signs</th></tr>
<tr><th>Somers' D</th><th>P-value</th><th>Somers' D</th><th>P-value</th><th>Somers' D</th><th>P-value</th><th>Somers' D</th><th>P-value</th></tr>
<tr><td colspan="2">AD-related Signs</td><td></td><td colspan="2"></td><td></td><td></td><td rowspan="9">0.811</td><td rowspan="9"><0.001</td></tr>
<tr><td>Hippocampus volume</td><td>0.801</td><td><0.001</td><td rowspan="3">0.809</td><td rowspan="3"><0.001</td><td colspan="2" rowspan="2"></td></tr>
<tr><td>Non-hippocampal AD signature region volume</td><td>0.788</td><td>0.02</td></tr>
<tr><td>Lobar micro-hemorrhages</td><td>0.773</td><td>0.28</td><td colspan="2"></td></tr>
<tr><td colspan="2">Vascular Signs</td><td></td><td colspan="2"></td><td></td><td></td></tr>
<tr><td>Log WMH volume</td><td>0.780</td><td>0.13</td><td colspan="2" rowspan="4">–</td><td rowspan="4">0.781</td><td rowspan="4">0.12</td></tr>
<tr><td>Cortical Infarcts</td><td>0.769</td><td>0.32</td></tr>
<tr><td>Lacunar Infarcts</td><td>0.768</td><td>0.99</td></tr>
<tr><td>Subcortical micro-hemorrhages</td><td>0.769</td><td>0.68</td></tr>
</table>

AD = Alzheimer’s disease, WMH = white matter hyperintensities. Somers D statistics were compared between the presenting model and the reference model. The reference model included: age, sex, race, education, APOE ε4, smoking, body mass index, hypertension, diabetes, total cholesterol level, and heart failure, with a Somers D statistic of 0.768.

© 2019 Wu A et al. *JAMA Network Open.*

**eFigure 1. Study Population Flow**

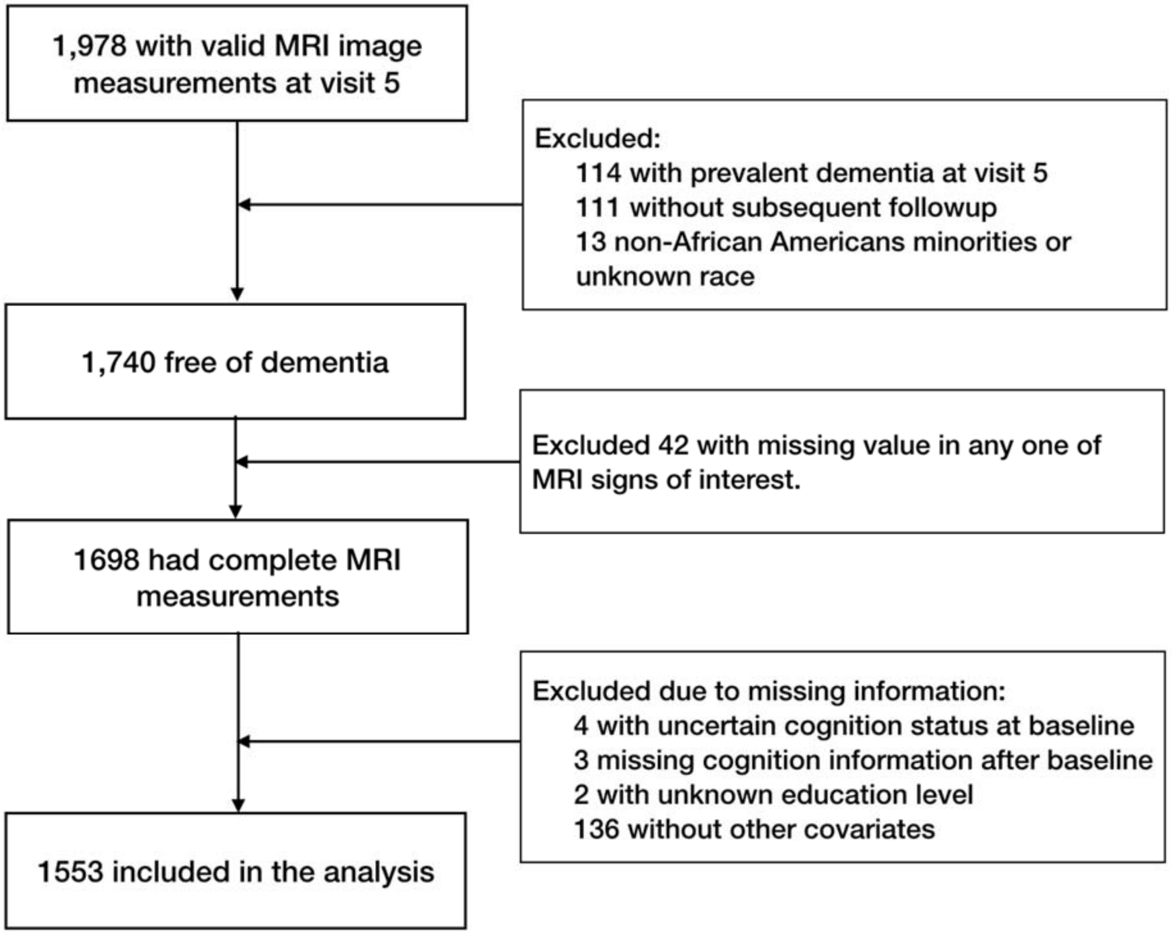


© 2019 Wu A et al. *JAMA Network Open.*

**eFigure 2. Dementia Risk by Number of Different Types of MRI Signs Among Participants With MCI**

AD = Alzheimer disease, MCI = mild cognitive decline
Hazard ratios and 95% confidence intervals of for incident dementia among participants with mild cognitive decline (MCI) were plotted. Model adjusted for age, gender, race, education, APOE ε4, smoking, body mass index, hypertension, diabetes, total cholesterol level, depressive symptoms, and heart failure. MRI signs of interest were three AD-related signs: low hippocampus volume, low non-hippocampal AD signature region volume (both were defined as a value below the lowest quartile), and having lobar micro-hemorrhages; and four vascular signs: high white matter hyperintensity volume (defined as a value above the median), having subcortical micro-hemorrhages, having cortical infarcts, and having lacunar infarcts. The categories labeled as “No Lesions”, “No AD Signs”, or “No Vascular Signs” were considered as normal referents.

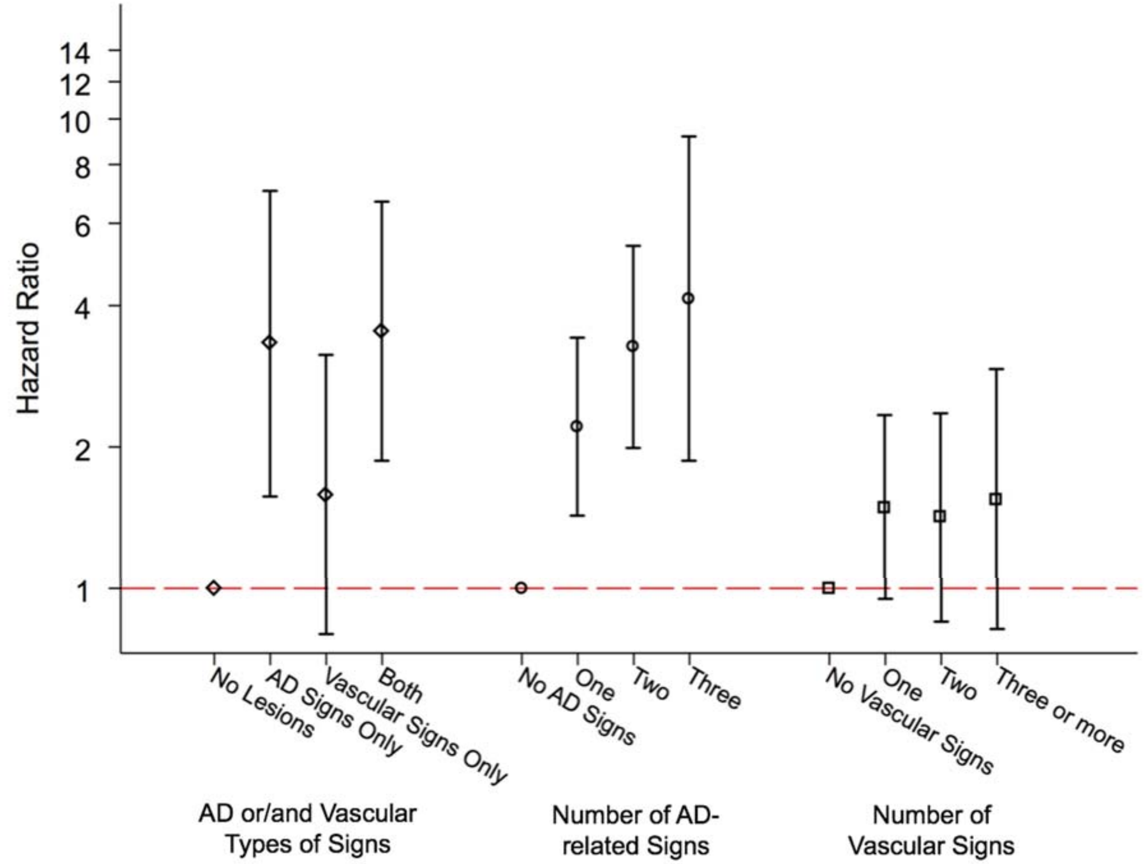


© 2019 Wu A et al. *JAMA Network Open.*

**eFigure 3. Adjusted Hazard Ratio of Incident Dementia Across Volumes of AD Signature Regions and WMH**

AD = Alzheimer disease, WMH = white matter hyperintensities

Hazard ratio of incident dementia across volume measurements of different types of brain lesions compared to the mean value (reference) of each measurement. Volume measurements (x-axis) were truncated at the 5th and 95th percentiles. The model was adjusted for age, gender, race, education, APOE ε4, smoking, body mass index, hypertension, diabetes, total cholesterol level, depressive symptoms, heart failure, and intracranial volume.

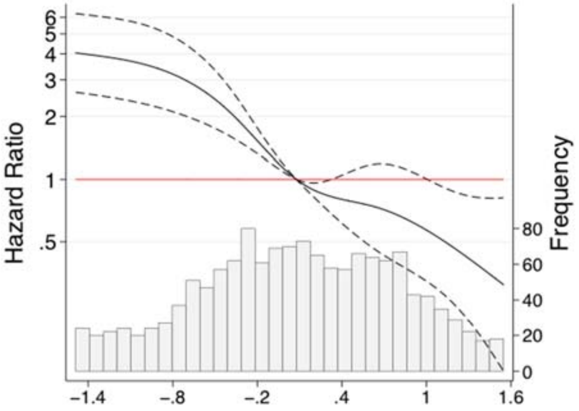


a. Standardized Hippocampus Volume

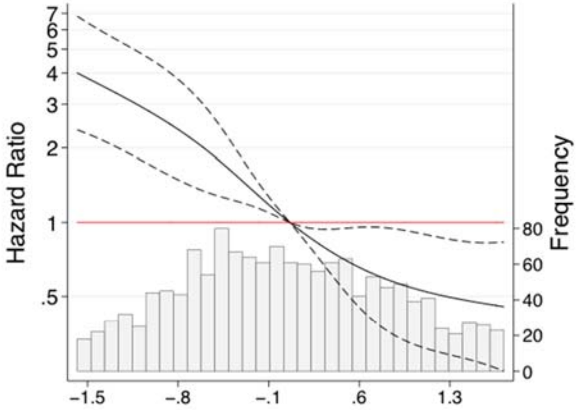


b. Standardized Non-hippocampus AD Signature Regions Volume

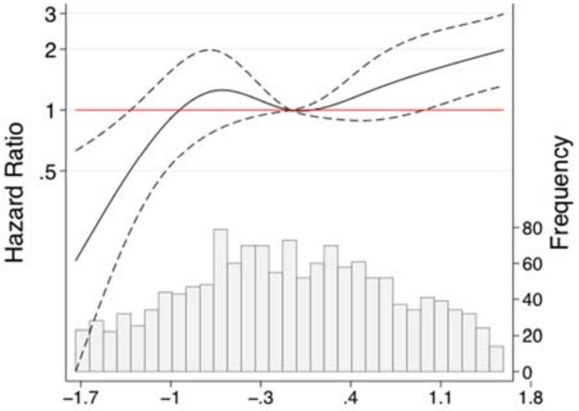


c. Standardized White Matter Hyperintensity Volume (log-scale)

© 2019 Wu A et al. *JAMA Network Open.*
